# Supplementary material for: Autophagic Survival Precedes Programmed Cell Death in Wheat Seedlings Exposed to Drought Stress
Source: Int J Mol Sci. 2019 Nov 16;20(22):5777. doi: 10.3390/ijms20225777 (PMC6888631; doi:10.3390/ijms20225777)
Supplement: Supplementary file 1 [file ijms-20-05777-s001.pdf]

Supplementary Material

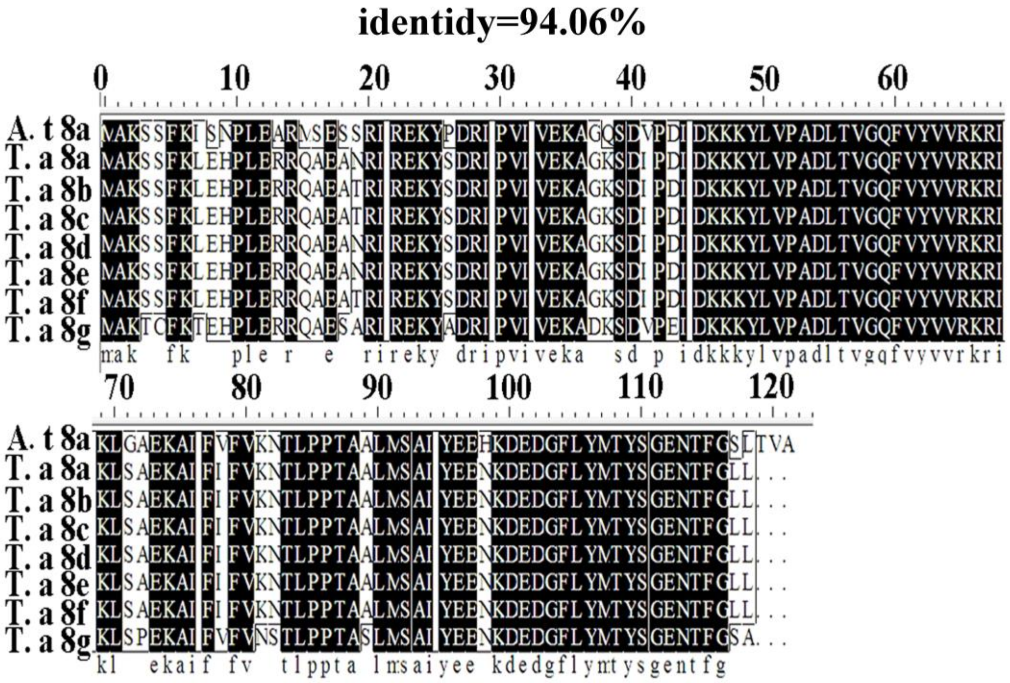

Figure S1 the protein sequence of *A. thaliana* APG8A was alignment with those of wheat ATG8 (ATG8a, 8b, 8c, 8d, 8e, 8f and 8g) by DNAMAN6.0 software.

Table S1 Primers used in the experiments

**Primer name**	**Sequence (5′-3′)**
ATG3QRTf	CCCTCCGATAAGCAGTTC
ATG3QRTr	TTTCACCAGCACCAAAGTA
ATG4QRTf	GAAAGCCCGCACAGAGTC
ATG4QRTr	ACCCGAGACCACATAGAGC
ATG5QRTf	GCTGCCTTCATCATAACTGG
ATG5QRTr	ATCGCATCTTCTAAATACTCG
ATG6QRTf	TTTCCGTCTCGGTCGTCT
ATG6QRTr	CAAACTTATGGCAAACTCG
ATG7QRTf	TGCCTCACTGGTGCTTAG
ATG7QRTr	CAATCCTTGAGTTGCCTTA
ATG8QRTf	AGGCTGATAAGTCTGATGTCC
ATG8QRTr	CGTCCTCGTCCTTGTTTT

ATG9QRTf	ACGGCAAACAGTTCTTAC
ATG9QRTr	TCTGCATGGTTACGGATA
ATG10QRTf	GACCCTGTCCCTGATGAT
ATG10QRTr	GAAGTGAGTGAGGAGGAAGAT
ATG12QRTf	ACAAGTTCAGGATTTCAGGACGAG
ATG12QRTr	TGCCGACAAAGCATAGTTTACCAC
ATG13QRTf	TGCTCCTGTGGTCATTC
ATG13QRTr	CCCGCTCATCCAGTTCAT
ATG16QRTf	CCAATGCGATGTATGAGGA
ATG16QRTr	GTAGATGGCACTGGTGTTTC
Atg6RNAiF	TCCATATACAAGACATCTAC
Atg6RNAiR	TTCGCTAAGGATGTTGTAGG
DsGFPF	ATGGTGAGCAAGGGCGAGG
DsGFPR	AGAAGATGGTGCGCTCCTG
α-TubulinF	AACTTCGCCCGTGGTCAT
α-TubulinR	CAGCGTTGAATACAAGGAATC

ATG(3,4….)QRT primers are used for Quantitative real-time reverse transcription-PCR (qRT-PCR) of *autophagy- related genes (ATGs)*. ATG6RNAi primers are used for amplifying the interference sequence of autophagy-related 6 (*ATG6*).
